# Clinical interview - how to establish a therapeutic relationship and effectively listen to the patient.

**DOI:** 10.1192/j.eurpsy.2023.185

**Published:** 2023-07-19

**Authors:** R. L. Medinas

**Affiliations:** Psychiatry, Centro Hospitalar de Lisboa Ocidental, Lisbon, Portugal

## Abstract

**Abstract:**

In this first session of our motivational interviewing workshop, we address the basic principles of how every therapeutic relationship should be established and the main differences for the right environment in which we can develop motivational interviewing tactics that can be used to precipitate enduring change.

**Disclosure of Interest:**

None Declared

